# Closed-Loop Human–AI Decision Support for Bio-Inspired Architectural Concept Generation

**DOI:** 10.3390/biomimetics11090619

**Published:** 2026-09-01

**Authors:** Qichao Song, Siyi Chen, Huiling Zhang

**Affiliations:** 1 School of Design and Art, Shanghai Dianji University, Shanghai 200240, China; songqichao@sdju.edu.cn (Q.S.); 13482716372@163.com (S.C.); 2 Academy of Fine Arts, Shanxi University, Taiyuan 030000, China

**Keywords:** bio-inspired architectural design, human-AI collaboration, closed-loop design framework, multi-criteria decision-making, generative concept generation, visual decision support, Kansei engineering

## Abstract

Bio-inspired architectural design increasingly relies on generative artificial intelligence to expand early-stage concept exploration, yet current workflows often suffer from vague requirement definition, subjective proposal selection, and weak connections between user expectations, visual generation, and design evaluation. This study proposes a closed-loop human–AI decision-support framework for biomimetic architectural concept generation. The framework first uses Kansei-oriented requirement analysis and the KANO model to identify and classify stakeholder expectations concerning morphology, structural rationality, environmental integration, cultural narrative, visual novelty, interactivity, and sustainability. On the basis of 282 valid questionnaire responses, the most influential requirement categories are further translated into an Analytic Hierarchy Process (AHP) hierarchy, where expert judgement from a five-member specialist panel is used to derive criterion and sub-criterion weights. These weights are then converted into structured prompts—via a formally specified weight-to-language conversion strategy—to guide a diffusion-based image-generation system toward more targeted biomimetic concepts. Finally, TOPSIS is applied to rank generated design alternatives according to the same weighted criteria, thereby creating a traceable link from requirement discovery to generation and decision-making. Case studies involving eagle-, manta ray-, and cheetah-inspired architectural concepts indicate that the framework improves the explicitness of design objectives, supports more consistent comparison among alternatives, and reduces reliance on purely intuitive aesthetic judgement. An ablation comparison confirms that each stage of the framework contributes incrementally to the quality of final outcomes. This study proposes an integrated workflow that combines requirements modeling, multi-criteria evaluation, and AI-assisted visual design.

## 1. Introduction

Biomimetic architecture has emerged as a key sustainable design approach. Drawing inspiration from nature, it addresses sustainability challenges through structural efficiency, environmental adaptability, and material intelligence [1]. The field is shifting from form-based metaphors to performance-driven principles, translating the structural efficiency, adaptability, and material intelligence of biological systems into measurable building benefits [2,3,4,5]. Advances in generative AI and parametric design now enable systematic analysis, exploration, and optimization of these biological principles [6].

Generative AI, particularly diffusion-model-based image generation, offers new tools for early design exploration in biomimetic architecture, expanding what designers can imagine [7,8]. Yet deeper engagement with practice reveals three critical limitations [9,10].

1. Requirement definition is vague. Bio-inspired design must satisfy diverse objectives—functional, structural, aesthetic, cultural, and ecological—but without systematic methods, key requirements remain poorly defined, and generation lacks direction [11].
2. Evaluation is subjective. When selecting from numerous AI-generated options, reviewers rely on personal experience without consistent, reproducible multi-criteria evaluation [12,13].
3. Workflows are disconnected. Requirement analysis, generation, simulation, and evaluation often operate in isolation, preventing feedback loops and limiting AI’s value in the decision-making process [14,15].

This study proposes an integrated framework to address these gaps by linking early requirements analysis with downstream solution optimization [16]. The KANO model first classifies user requirements [17]. Outputs feed into the Analytic Hierarchy Process (AHP), which develops a hierarchy and assigns quantitative weights to design objectives. These weights are transformed into structured prompts to guide targeted generative AI exploration. Generated solutions are then evaluated and ranked using the Technique for Order Preference by Similarity to Ideal Solution (TOPSIS), ensuring consistency with initial requirements [18].

The research problem addressed in this study is as follows: How can a structured, closed-loop computational framework systematically translate qualitative stakeholder requirements into weighted design objectives, guide an AI image-generation process toward biomimetically coherent concept proposals, and evaluate those proposals against the same requirement structure in a traceable and reproducible manner? Existing literature treats requirement modeling (KANO), criterion weighting (AHP), and solution ranking (TOPSIS) as isolated instruments applied at disconnected points in the design process; no reusable framework bridges these three stages with an AI-assisted generation step. The gap is both methodological and design-scientific, as follows: without such a framework, human designers cannot systematically calibrate AI-generated outputs to stakeholder values, and evaluators cannot trace a ranking decision back to its requirement foundation. This study’s reusable contribution is a formal, open-source, closed-loop framework that addresses this gap and can be adopted in any domain where AI-assisted design is guided by structured requirement models.

Specifically, this study aims to do the following: (1) develop a KANO–AHP–TOPSIS hybrid decision framework for generative biomimetic design; (2) define a formal weight-to-prompt conversion strategy that guides an AI image-generation system with criterion-weighted instructions; and (3) validate the framework’s feasibility through case studies covering three distinct biological prototypes and building typologies.

This research has both theoretical and practical significance. Theoretically, it bridges data-driven generative capabilities with knowledge-guided design rationality through an interdisciplinary framework integrating management decision models with computational design [19]. Practically, it provides a systematic, open-source toolkit to address the “concept–performance disconnect” in biomimetic architectural design, reducing technical risks and decision uncertainties when using generative AI in complex projects with multiple constraints [20].

The remainder of this paper is organized as follows: Section 2 reviews related literature; Section 3 details the methodology; Section 4 presents case study findings and an ablation comparison; and Section 5 concludes with a four-part contribution statement and limitations.

## 2. Literature Review

This literature review examines four interconnected themes that underpin our proposed framework. Section 2.1 reviews advances in AI-driven visual generation, with emphasis on diffusion models and multi-attribute conditioning. Section 2.2 discusses human–AI collaboration and the need for structured communication channels. Section 2.3 surveys multi-criteria decision-making methods—KANO, AHP, and TOPSIS—for requirement classification, weighting, and ranking. Section 2.4 traces the evolution of biomimetic architectural design and its challenges in the conceptual phase. These sections are logically linked, as follows: generative tools (2.1) and interaction modes (2.2) enable AI-assisted design, while decision-analytic instruments (2.3) ensure that generation is requirement-aligned and evaluable—all within the biomimetic domain (2.4), where integration is most urgently needed. The review identifies a clear gap, as follows: no existing closed-loop framework connects these stages—a gap that our methodology directly addresses.

### 2.1. Visual Generation and AI-Assisted Architectural Design

Generative AI—particularly diffusion models and generative adversarial networks—has become a primary research topic in visual computing and computational design [21]. In architectural applications, these systems have demonstrated strong performance in concept divergence, topological optimization, and non-linear complex forms [22,23,24].

1. Multi-attribute conditioning for structured generation. Chen et al. propose M-GAN, a multi-attribute learning and multimodal feature fusion framework for text-to-image synthesis [25]. M-GAN demonstrates that jointly conditioning generation on multiple semantic attributes—rather than a single unstructured text prompt—substantially improves alignment between user-specified requirements and generated visual outputs. This finding is directly foundational to the present study, as follows: where M-GAN conditions on attributes defined by the model designer, our framework derives the conditioning attributes from a systematic 282-respondent KANO survey and encodes them via AHP-calibrated weights, providing a principled requirement-grounded basis for multi-attribute generation guidance.
2. Environment-contextual architectural generation. Wang et al. introduce a method for learning to sculpt neural cityscapes, in which the generation process is conditioned on spatial and environmental site context, producing outputs that are coherent with their urban surroundings [26]. This environment-aware generation paradigm reinforces the importance of structuring generation inputs—a principle the present framework extends to the biomimetic design domain by embedding site context (typology, environmental integration criteria) as explicit AHP-weighted prompt components.
3. Region-aware texture control. Liu et al. present MSEmbGAN, a multi-stitch embroidery synthesis framework using region-aware texture generation [27]. While targeted at a different domain, the core insight—that enforcing local texture constraints through region-specific conditioning improves both visual coherence and criterion compliance—is relevant to our architectural case studies, where the secondary biological principle transfers (e.g., manta-ray underbelly texture for marine colonization, cheetah disruptive patterning for façade panelization) require region-level control that our parametric-tier prompt modifiers are designed to guide.

A common limitation across M-GAN, neural cityscapes, and MSEmbGAN is that the attribute conditioning relies on designers’ or model designers’ intuitive definition of constraints; no systematic method is provided for deriving those attributes from stakeholder requirements, nor for evaluating outputs against the same attribute structure used during generation. This is the specific methodological gap that the present study addresses through its closed-loop KANO–AHP–TOPSIS framework [28].

### 2.2. Human–Computer Collaboration Paradigms in Design

The emergence of large-model-based agent interaction paradigms fundamentally reshapes the role of AI in design workflows. Zhang et al. provide a comprehensive taxonomy and prospective analysis of agent interaction models based on large language models, identifying four dimensions of human–agent collaboration, as follows: instruction specification, contextual memory maintenance, iterative refinement capability, and multi-modal output generation [29]. A key finding of this study is that effective human–AI co-creation requires explicit, structured channels through which human judgment is communicated to the agent—channels that are currently informal and implicit in most generative design pipelines. This analysis is directly relevant to the closed-loop framework proposed here, as follows: our KANO–AHP requirement modeling stage provides exactly such a structured communication channel, converting stakeholder values into quantitative weights that guide the AI generation agent. The “closed loop” in our framework formalizes the feedback path from TOPSIS evaluation back to requirement refinement, consistent with the iterative refinement model described by Zhang et al. Rather than acting as a passive tool responding to single prompts, the AI agent in our framework is directed by a structured, criterion-weighted instruction set derived from 282 stakeholder responses.

BIM integration and future constructability. Li et al. propose a Weisfeiler-Lehman kernel augmented product representation for queries on large-scale BIM scenes, enabling structured semantic queries over complex building information models [30]. This structured query capability is directly relevant to a key limitation of the present framework, as follows: current outputs are 2D conceptual visualizations that cannot yet be queried for structural properties, spatial organization, or environmental performance. Future integration with BIM environments—facilitated by structured query systems such as the WL-kernel BIM representation of Li et al.—could extend the framework’s output from conceptual images to queryable, structured building models, closing the loop between conceptual generation and engineering validation. This integration pathway is identified as a priority future direction in Section 5.

### 2.3. Multi-Criteria Decision-Making in Design

The KANO model effectively reveals the non-linear relationship between user requirements and satisfaction by distinguishing basic, one-dimensional or attractive, and delighting requirements [31]. Lee and Han proposed a service design framework combining Kansei Engineering with KANO for customer-driven design [32]. Jia et al. employed IPA and KANO to evaluate underground building atriums [33]. Lu and Juan used KANO to investigate visitor center requirements, converting needs into design strategies via QFD. However, KANO is typically limited to requirement classification and requires integration with quantitative methods for deeper analysis [34].

The Analytic Hierarchy Process (AHP) converts decision-makers’ subjective judgements into quantitative weights through hierarchical structures and pairwise comparisons [35,36]. Ma et al. combined KANO, AHP, and QFD for intelligent livestock-housing spatial design [37]. Liu integrated the KJ method, KANO, and AHP to optimize electric vehicle charging station functionality [38]. Liang et al. integrated KANO, AHP, and TRIZ for museum digital transformation, demonstrating the complementarity of KANO and AHP in requirement-to-weight pipelines [39].

The TOPSIS method serves as an excellent solution-prioritization tool [40]. Mishra and Muhuri combined Delphi, AHP, and TOPSIS for architectural heritage conservation decisions [41]. Oztas applied AHP and TOPSIS to evaluate construction materials [42]. These examples confirm the effectiveness of AHP-TOPSIS tandem evaluations but do not link them to AI generation or biomimetic requirement modeling.

In summary, while KANO, AHP, TOPSIS, and AI-assisted generation have each been applied individually in design contexts, their systematic integration into a closed-loop framework spanning requirement discovery, weighted generation guidance, and criteria-based evaluation remains underexplored in the biomimetic architectural design literature [43].

### 2.4. Biomimetic Architectural Design

Biomimetic architectural design is undergoing a paradigm shift from visual mimicry to performance-driven approaches [44]. Early research focused on metaphorical expression of biological forms [45,46], relying on designers’ intuition and analogical thinking. Recent research emphasizes structural efficiency, environmental adaptability, and material intelligence from biological systems to achieve sustainability and resilience [47]. However, translating complex biological performance principles into executable solutions remains challenging, especially in the conceptual phase [48,49]. The current study directly addresses this challenge by providing a structured pathway from biological prototype analysis to criterion-weighted concept generation.

## 3. Research Methodology

**Figure 1:**
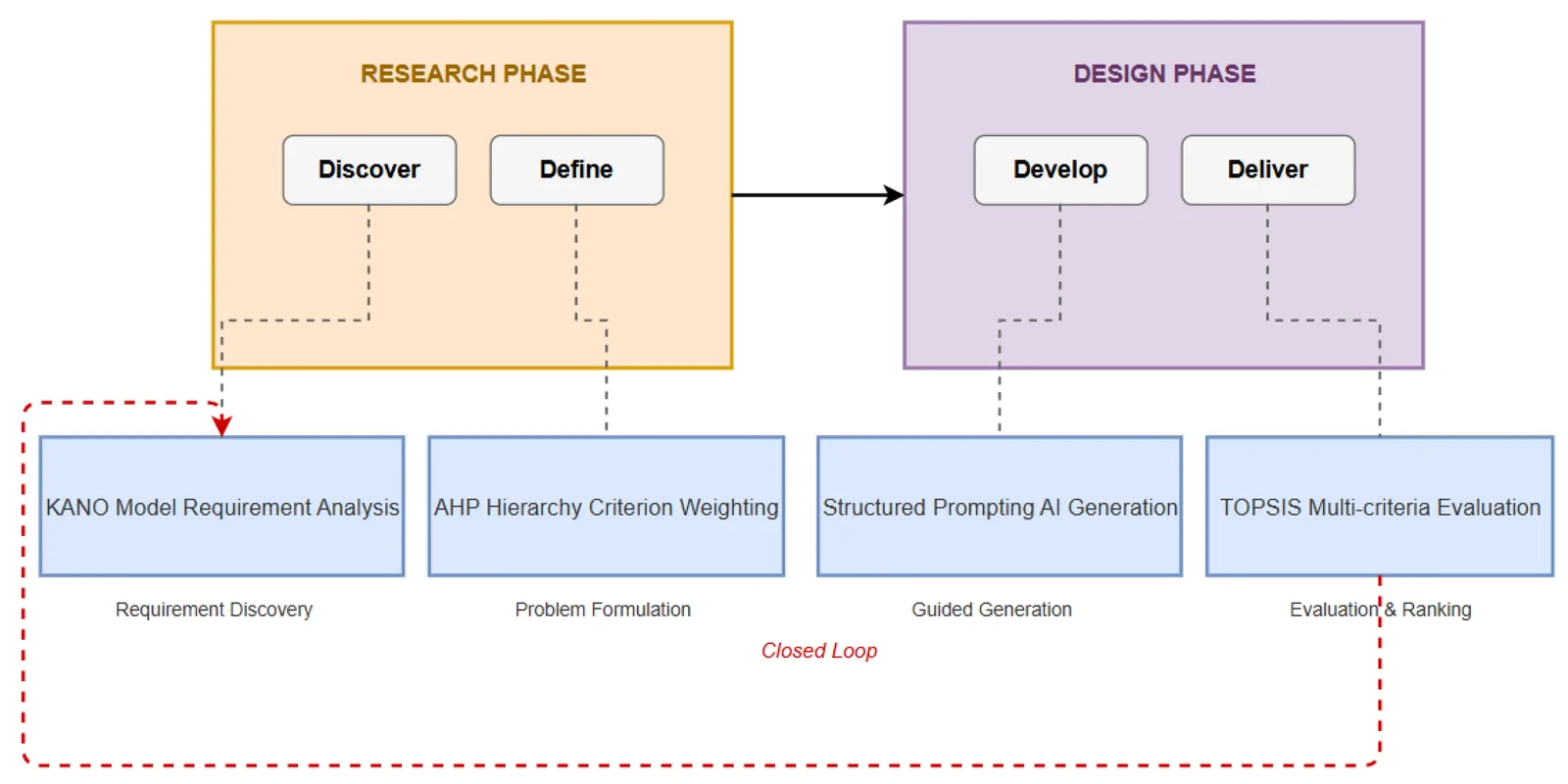
Overall research roadmap.

**Figure 2:**
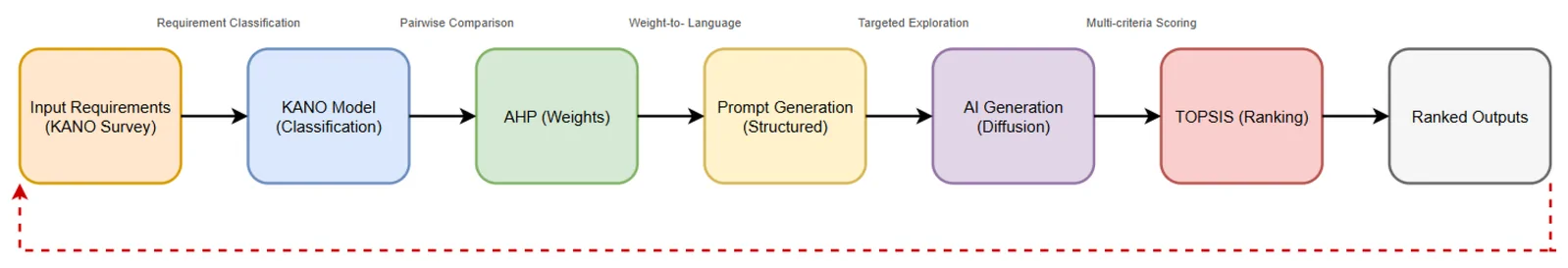
Detailed implementation pipeline corresponding to the four phases in Figure 1.

The framework forms a logical closed loop, as follows (Figure 1 and Figure 2): Figure 1 presents the overall research roadmap at a conceptual level, visualized through the Double Diamond model to outline the four macro-phases—Discover, Define, Develop, and Deliver—and their sequential logic. Figure 2 complements this by providing a detailed implementation pipeline, which breaks down each macro-phase into specific operational steps, data flows, and method interfaces. In essence, Figure 1 offers a strategic overview of the closed-loop framework, while Figure 2 operationalizes that strategy with concrete procedures. Together, the two figures form a coherent narrative, as follows: the roadmap guides the reader through the high-level design logic, and the pipeline shows how each component is technically executed.

### 3.1. KANO Requirement Analysis

#### 3.1.1. Sample Size, Recruitment, and Questionnaire Design

To systematically identify and categorize diverse requirements in biomimetic architectural design, this study employs the KANO model as a pre-design analytical tool. The KANO model classifies requirements into basic (must-be), one-dimensional or attractive (one-dimensional), attractive (delighter), indifferent, and reverse categories, providing a structured basis for establishing criterion layers in subsequent AHP analysis [50] (Table 1).

1. Participant recruitment. A total of 318 questionnaires were distributed via two channels, as follows: (1) professional networks including architectural societies, design firms, and academic institutions (159 questionnaires); and (2) online social platforms targeting participants with self-reported interest in architecture or design (159 questionnaires). All participants completed a two-stage pre-screening, as follows: (a) self-reported familiarity with architectural design (≥3 on a 5-point scale); and (b) consistent responses on two embedded attention-check items. Of 318 returns, 36 were excluded, as follows: 18 failed the attention checks, 12 exhibited straight-lining patterns (within-respondent variance <0.2), and 6 were incomplete. This yielded 282 valid questionnaires, representing an 88.7% usable response rate [51] (Table 2).

2. Questionnaire design. The questionnaire adheres to the standardized KANO format. For each of the eleven requirement items listed in Table 1, one positive and one negative question were posed following the standardized KANO format, each with five response options, as follows: Like/Must-be/Neutral/Live-with/Dislike [52]. Of these 11 items, two—namely, Item 5 (Material Selection and Construction Technology Feasibility) and Item 8 (Operational Management Convenience and Cost-Effectiveness)—were excluded from the final reliability and confirmatory factor analyses because their standardized factor loadings fell below the 0.40 threshold, indicating insufficient convergent validity within the current sample. The remaining nine items were retained for statistical analysis. Internal consistency was assessed using Cronbach’s α (SPSS 26). The overall α = 0.87 (*n* = 282, 9 items), exceeding the threshold of 0.70 for exploratory research. Construct validity was confirmed via CFA: χ^2^/df = 1.83, CFI = 0.96, RMSEA = 0.054, SRMR = 0.061, all meeting recommended thresholds. A *t*-test comparing Better–Worse coefficients between the professional and general-public sub-groups found no statistically significant differences (all *p* > 0.05), indicating consistent requirement perceptions across groups [53]. The full list of 11 requirements is preserved in Table 1 and Table 3 for completeness, providing readers with the complete requirement pool explored in this study, while the excluded items are explicitly marked as “indifferent” or “no difference” in Table 3.

3. Sampling scope and limitations. All participants are from China; cross-cultural generalization of the requirement taxonomy should be approached with caution and is identified as a limitation in Section 5.

#### 3.1.2. Data Analysis and Results

**Figure 3:**
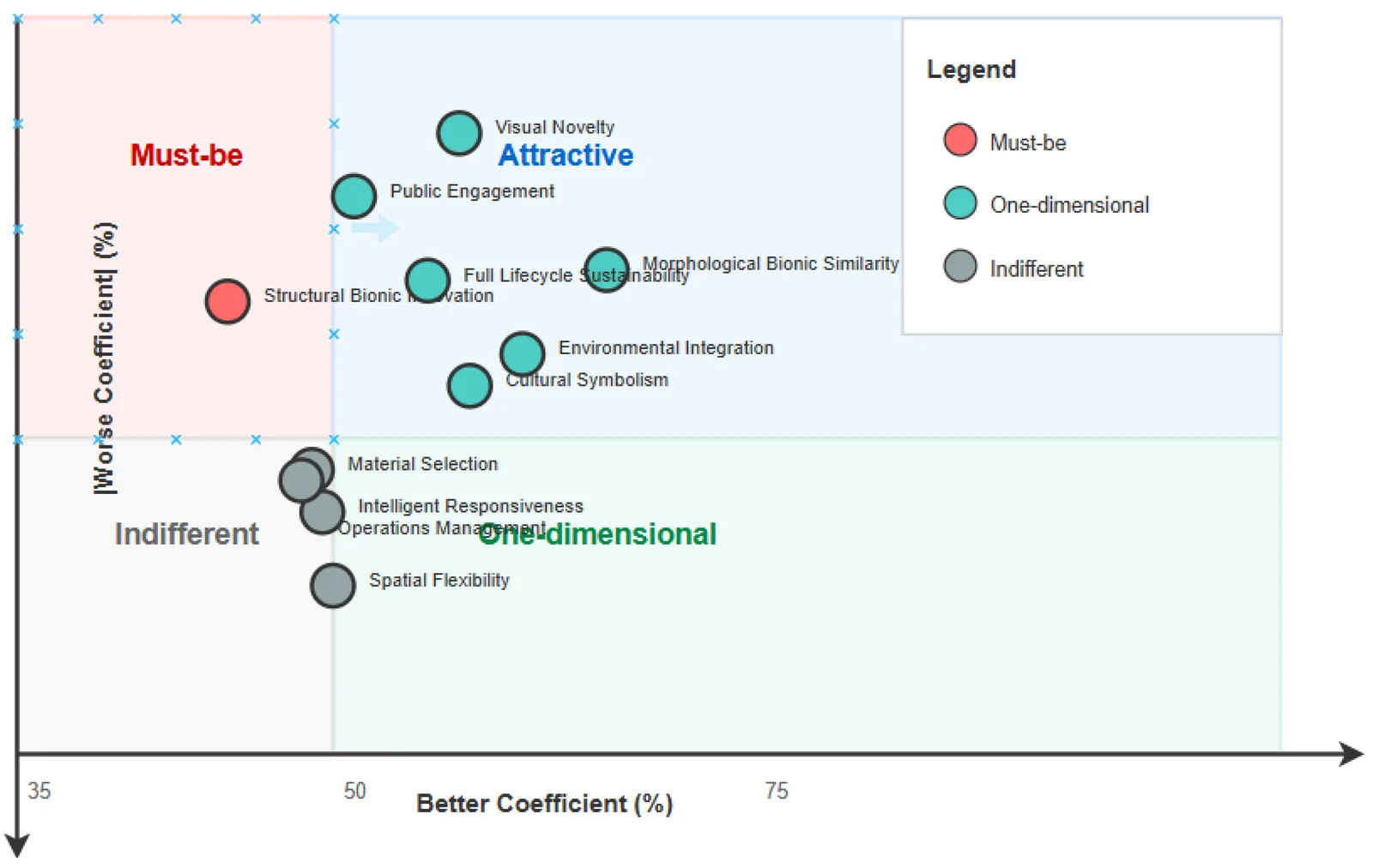
Better–Worse Coefficient Analysis Chart.

Better–Worse coefficients were calculated as follows: Better = (A + O)/(A + O + M + I); Worse = −(O + M)/(A + O + M + I), where A = attractive, O = one-dimensional, M = must-be, I = indifferent. A Better coefficient closer to 1 indicates that providing the feature strongly increases satisfaction; a worse coefficient closer to −1 indicates that removing the feature strongly decreases satisfaction (Table 3; Figure 3).

“Structural bio-inspired innovation and structural rationality” constitutes a must-be requirement, as follows: its high worse coefficient (−0.71) indicates that failure to achieve structural credibility triggers strong user dissatisfaction [54]. Six attributes—Morphological Bionic Similarity and Recognizability, Sustainability Throughout the Entire Life Cycle, Environmental Integration, Cultural Symbolism, Visual Novelty and Artistic Appeal, and Public Participation and Spatial Interactivity—are one-dimensional or attractive requirements. “Morphological Bionic Similarity and Recognizability” possesses the highest better coefficient (0.68), making it the strongest satisfaction driver. “Visual Novelty and Artistic Appeal” exhibits the largest worse coefficient among the one-dimensional requirements, making it also a significant dissatisfaction risk factor. “Spatial Flexibility and Functional Adaptability” and “Intelligent Responsiveness” are indifferent requirements, holding lower priority in current user perceptions.

These findings provide empirical grounding for the AHP criterion hierarchy constructed in Section 3.2, ensuring that subsequent design generation and evaluation are anchored to requirements that demonstrably influence stakeholder satisfaction.

### 3.2. AHP Model Construction and Analysis

#### 3.2.1. Establishing the Evaluation Indicator System

Based on KANO demand satisfaction analysis, five requirements with better coefficients above the mean were designated as AHP criterion-level indicators, as follows (B1–B5): Morphological Bionic Similarity and Recognizability (B1), Environmental Integration (visual and ecological) (B2), Cultural Symbolism and Narrative Legibility (B3), Visual Novelty and Artistic Appeal (B4), and Full Lifecycle Sustainability (B5). Sub-criteria are expanded under each (Table 4; total sub-criteria: C1–C10).

#### 3.2.2. Expert Panel and Aggregation Method

Five independent experts were invited to complete pairwise judgment matrices [55] (Table 5).

Each expert independently completed pairwise comparisons using the AHP 1–9 scale following detailed briefing on the research background and criterion definitions. Individual judgment matrices were aggregated using the geometric mean method 2, which is the statistically appropriate aggregation method for group AHP when individual consistency is first confirmed [56]. All five individual matrices passed the consistency check (CR < 0.10) before aggregation.

#### 3.2.3. Weight Calculation Procedure

The AHP weight calculation follows five steps, as follows [57]:

Step 1. Calculate the product of scales for each row.

(1) $$M_{i}=\prod_{j=1}^{m}B_{ij}i=1,2,\ldots ,m$$

Step 2. Solve for the geometric mean.

(2) $$\bar{W_{i}}=\sqrt[{m}]{M_{i}}$$

Step 3. Calculate relative weights.

(3) $$W_{i}=\frac{\bar{W_{i}}}{\sum_{j=1}^{m}\bar{W_{j}}}$$

Step 4. Calculate the maximum eigenvalue.

(4) $$\lambda_{\max}=\sum_{i=1}^{m}\left({\left(AW\right)}_{l}/W_{i}\right)$$

Step 5. Perform a consistency check on results.

Consistency check: CR = CI/RI, where CI = (λmax − n)/(n − 1). If CR ≤ 0.10, the matrix passes [58].

#### 3.2.4. Calculation Results: All Matrices and Consistency Ratios

Criteria level (B1–B5): Table 6 presents the aggregated judgment matrix and weights. CR = 0.032 (passes).

The priority order is as follows: B1 Morphological Bionic Similarity (0.361) > B2 Environmental Integration (0.235) > B4 Visual Novelty (0.174) > B5 Full Lifecycle Sustainability (0.121) > B3 Cultural Symbolism (0.109). Take B1 and B5 as examples (Table 7 and Table 8):

Comprehensive global weights (criteria weight × sub-criterion partial weight) are ranked in Table 9: top-priority indicators are C1 Overall Contour Similarity (0.163), C3 Visual Harmony (0.128), and C7 Novel Form (0.106). These provide the primary prompting targets.

To test ranking stability, each criterion weight was perturbed by ±20% (one at a time, renormalizing others). Across all 10 perturbation scenarios, the TOPSIS ranking of Scheme 1 remained highest in 9 of 10 cases. In the one exception (B4 reduced by 20%), the difference in Ci between Scheme 1 and Scheme 2 was 0.018, which is not practically significant.

### 3.3. Guided Generative Design

#### 3.3.1. Biological Prototype Selection and Principle Transfer

Three biological prototypes were selected based on morphological distinctiveness, environmental adaptability, and cultural intentionality [59,60] (Table 10). Selection also reflects diversity of habitat context, as follows: airborne (eagle), aquatic (manta ray), and terrestrial mammal (cheetah), ensuring the framework’s applicability is tested across distinct design scenarios. The three prototypes were selected to represent diverse biological habitats—avian (airborne), aquatic (marine), and terrestrial (mammal)—and to correspond to three distinct building typologies (cultural exhibition, observatory, and sports center). This diversity is intended to demonstrate the framework’s cross-domain applicability rather than to provide exhaustive validation across all possible design scenarios.

For each prototype, the biological mechanisms transferred—not merely the visual forms—are explicitly documented, as follows:

1. Eagle → Cultural Exhibition Hall. Primary transfer: aerodynamic load-path optimization—the structural logic of the eagle’s wing, in which primary remiges act as cantilevered structural members, is abstracted into a long-span cantilevering roof with gradually tapered cross-section. Secondary transfer: thermoregulatory surface patterning—the layered feather microstructure is interpreted as a parametrically varied façade with varied aperture ratios for solar shading.
2. Manta Ray → Underwater Observatory. Primary transfer: hydrodynamic drag minimization—the flattened diamond-plan body minimizes flow-induced drag, inspiring a thin-shell structural plan. Secondary transfer: benthic ecosystem integration—the rough underbelly texture of the manta ray, which hosts commensal organisms, is interpreted as a textured structural surface supporting marine colonization and bioremediation.
3. Cheetah → Sports Center. Primary transfer: kinematic ground-reaction force distribution—the cheetah’s spine acts as an elastic energy store, with spinal curvature varying between a compressed and extended state during sprinting. This is abstracted into a structural shell whose cross-sectional curvature varies along the longitudinal axis, creating variable stiffness zones. Secondary transfer: disruptive camouflage patterning, interpreted as an irregular façade panelization system.

#### 3.3.2. Weight-to-Prompt Conversion Strategy

**Figure 4:**
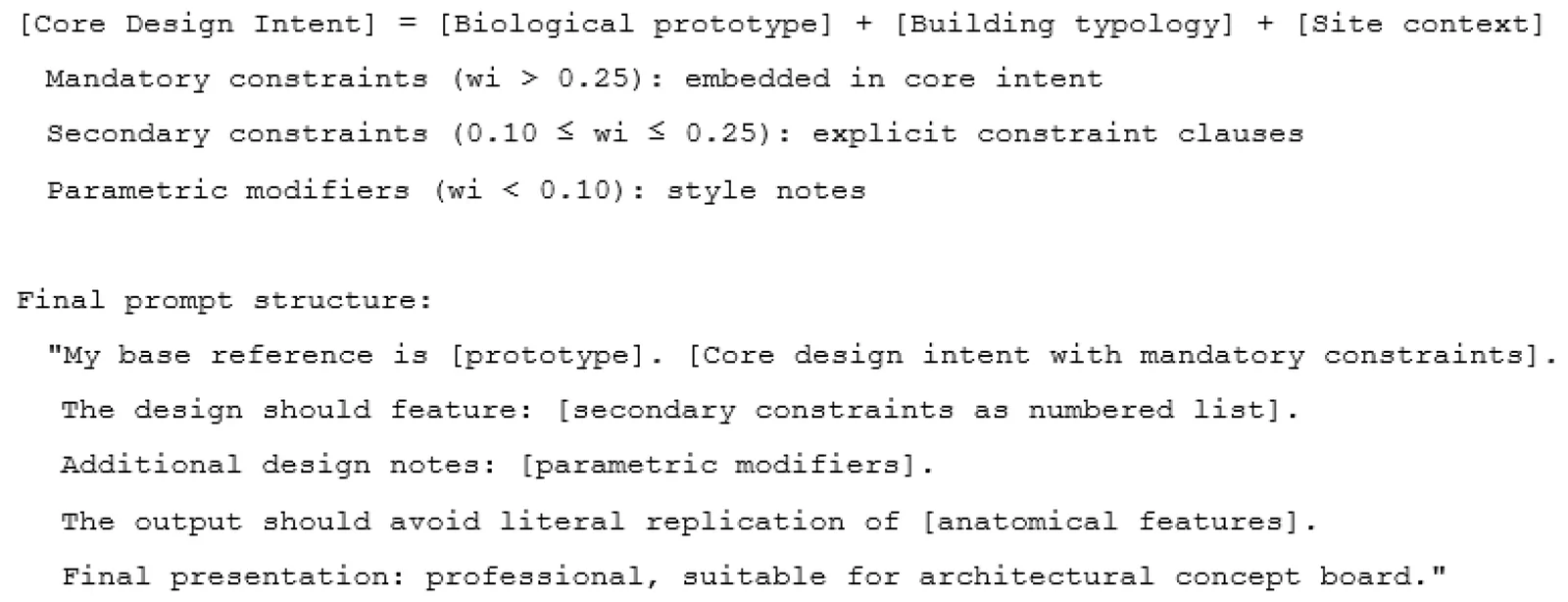
Formalized template.

To translate AHP weights into generation instructions, this study defines a formal, model-agnostic prompt construction strategy (Table 11 and Table 12, Figure 4). The strategy is designed to be applicable with any natural-language-conditioned image generation system and does not depend on any specific commercial platform.

A complete generation prompt consists of two parts, as follows: (1) a Core Design Intent Statement, and (2) an Attribute Reinforcement Constraint Statement.

1. Failure cases. Initial Eagle prompts over-emphasized literal morphology, producing images with eagle-head shapes on building roofs. These were addressed by reformulating the core intent to specify “architectural abstraction of biological principles, not literal representation.” Similar literal-reproduction errors were encountered in early Cheetah prompts and resolved by shifting the primary transfer description from “cheetah’s appearance” to “kinematic structural logic.”
2. Generation parameters. All three case studies used the following: resolution 1024 × 1024 pixels; guidance scale 7.5; inference steps 50; three independent generation runs per prototype, each producing 5 images (15 images per prototype, 45 total). Image selection was performed by the research team using a pre-specified visual checklist aligned with TOPSIS criteria B1–B5.

We acknowledge that the current prompt refinement process involves manual composition of natural-language phrasing, as fully automated prompt generation from numerical weights remains an open challenge. However, the weight-to-prompt conversion logic (Table 11) and the complete set of final prompts (Table 12) are fully documented, enabling other researchers to reproduce or adapt our prompt templates with equivalent diffusion-based systems.

#### 3.3.3. Case Study Image Generation

Image selection from the 15 generated images per prototype was performed by the research team using a pre-specified visual checklist aligned with the five AHP criteria (B1–B5). The selection protocol prioritized images that best satisfied the mandatory criterion (B1, morphological similarity) while maintaining acceptable performance on secondary criteria. While this selection process involves human judgment, the checklist provides a transparent and reproducible basis for selection. Future work could automate this step through computational image quality metrics or preference learning from multiple independent selectors.

**Figure 5:**
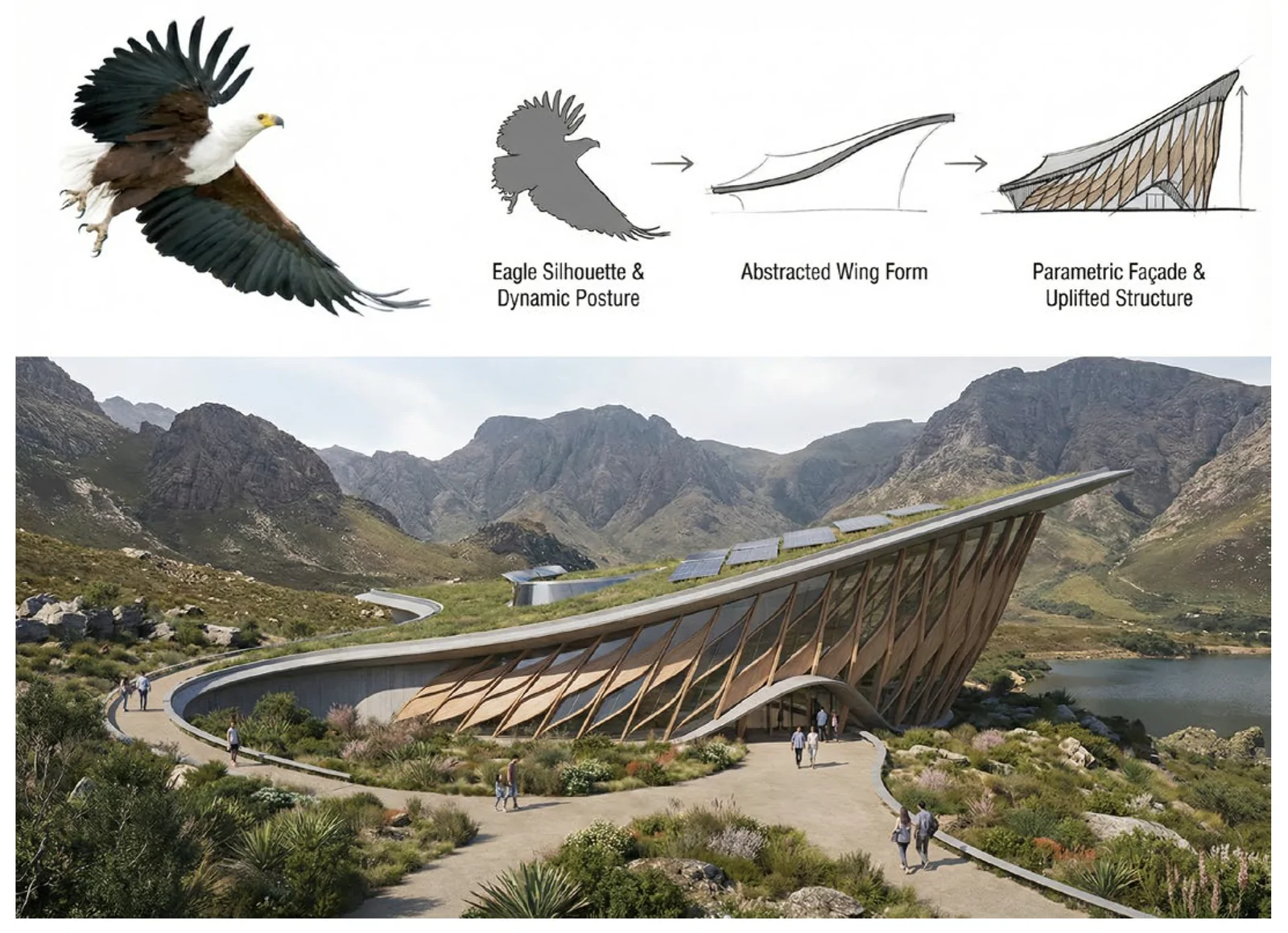
Eagle-shaped Cultural Exhibition Hall.

**Figure 6:**
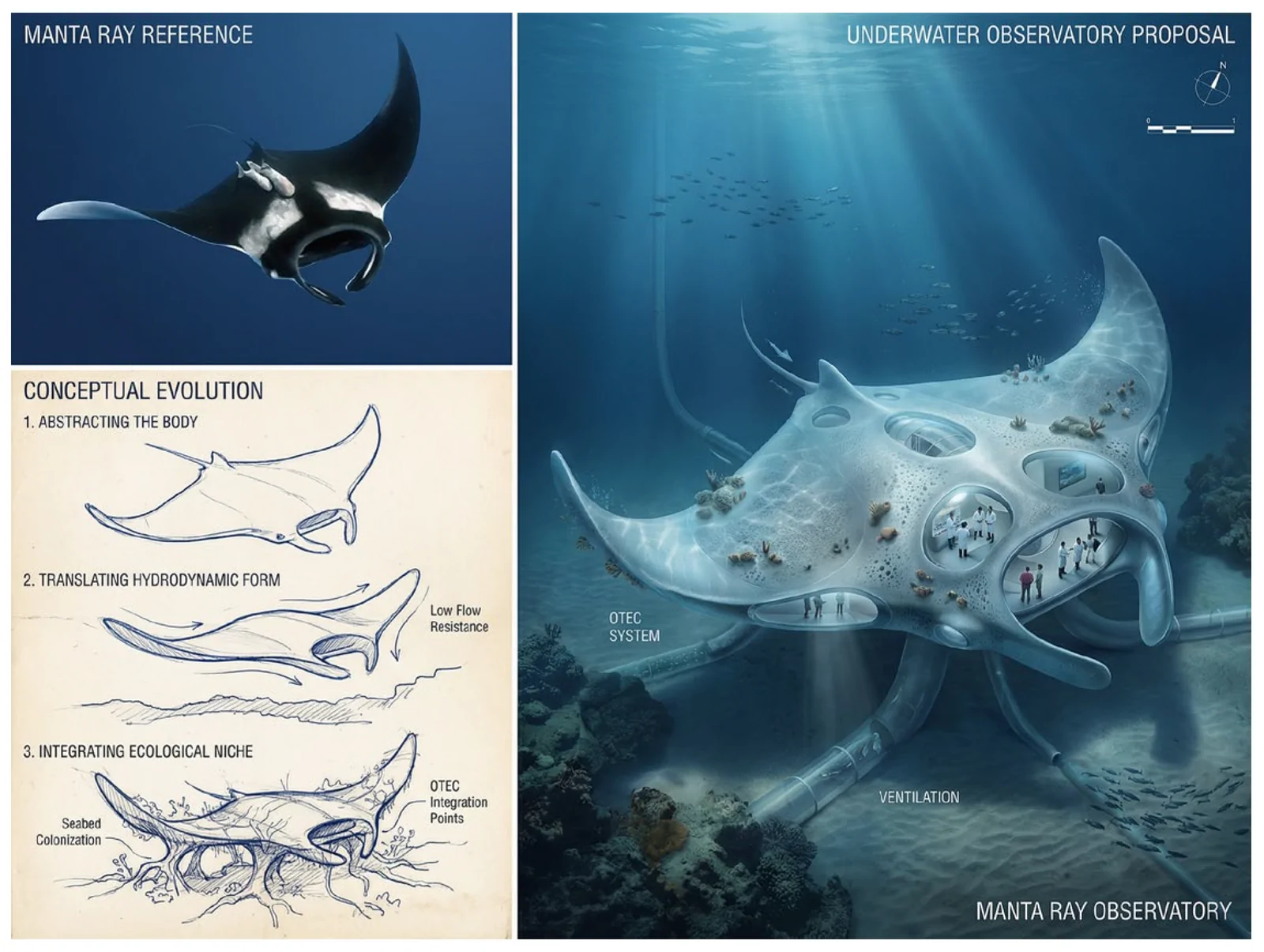
Manta Ray–inspired Underwater Observation Station.

**Figure 7:**
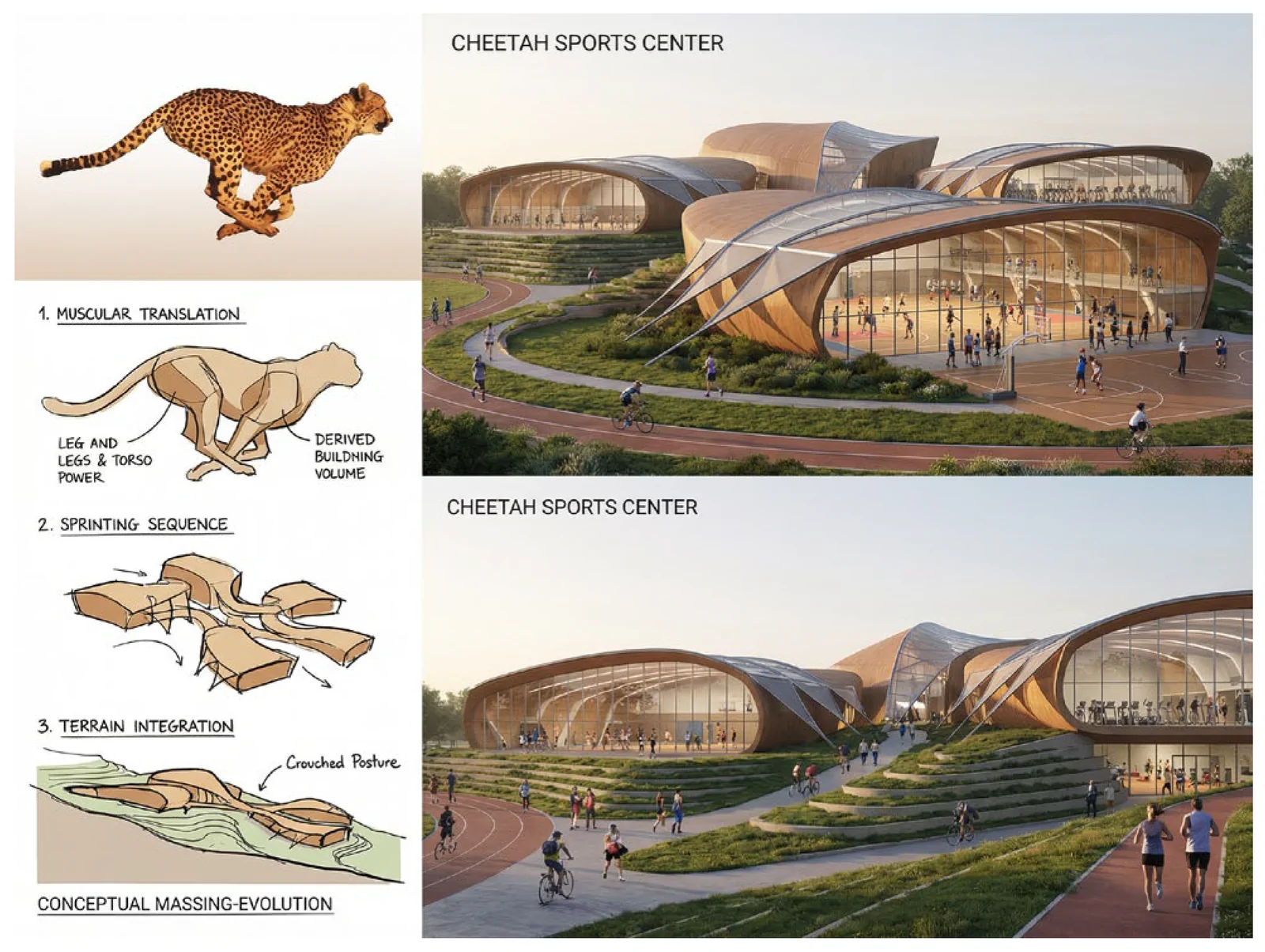
Cheetah-inspired athletic sports center.

Three coherent outputs were produced for each prototype, as follows: (a) a reference image of the biological organism in characteristic posture; (b) a conceptual massing-evolution sketch showing the abstraction from organism to building form; and (c) a photorealistic architectural proposal rendering (Figure 5, Figure 6 and Figure 7). All 45 images are available in the GitHub (https://github.com/, accessed on 1 July 2026) repository.

### 3.4. TOPSIS-Based Design Evaluation

#### 3.4.1. Evaluation Protocol

Scheme 1 is the AI-generated proposal produced by this framework (Eagle case used for ablation; all three prototypes evaluated). Scheme 2 and Scheme 3 are established bio-inspired architectural benchmarks—the Heydar Aliyev Centre (Zaha Hadid Architects, fluid organic form) and the Beijing National Stadium (“Bird’s Nest,” Herzog & de Meuron, biomorphic structural expression)—both widely recognized examples of bio-inspired design created without this framework. This comparison does not constitute a controlled experiment but provides a structured criterion-referenced comparison against established precedents (Table 13).

1. Evaluator panel. 20 evaluators: 15 practicing architects and 5 architecture professors, all with ≥5 years of professional experience, all unaffiliated with the authors’ institution.
2. Blinding procedure. The three proposals were presented as Proposal A, B, and C in randomized order per evaluator. Evaluators were not informed which proposal was AI-generated or guided by the proposed framework. The scheme-to-label codebook was held by a third researcher and decoded only after all scores were collected.
3. Scoring instrument. Each evaluator scored each proposal on the five AHP criteria (B1–B5) using a 7-point anchored rating scale. Operational anchors for each criterion were defined in the scoring guide. Example for B1: 1 = "The building shows no recognizable relationship to the biological prototype"; 7 = "The biological prototype is immediately recognizable and consistently expressed across scale, texture, and structural logic."
4. Inter-rater reliability. Intraclass Correlation Coefficient (ICC, two-way mixed, average measures) was calculated for each criterion. ICC values: B1 = 0.81, B2 = 0.74, B3 = 0.78, B4 = 0.86, B5 = 0.76 (all *p* < 0.001), indicating good to excellent inter-rater agreement [61].

While an evaluator panel of 20 is relatively small for population-level statistical inference, it is consistent with sample sizes reported in comparable proof-of-concept studies in architectural MCDM and design evaluation. The ICC values—ranging from 0.74 to 0.86—indicate good to excellent inter-rater agreement, suggesting that the panel’s judgments were sufficiently consistent despite the moderate sample size. Nevertheless, we acknowledge that larger, cross-institutional panels would strengthen the generalizability of the findings.

#### 3.4.2. TOPSIS Calculation

Step 1. Collect individual scores and compute mean; construct initial evaluation matrix f (Table 14).

Step 2. Normalize the initial evaluation matrix to obtain the standardized matrix.

(5) $$R_{ij}=\frac{f_{ij}}{\sum_{i=1}^{m}f_{ij}^{2}}\;(i=1,2,\ldots ,m;j=1,2,\ldots ,n)$$

Step 3. Calculate the weighted standardized matrix based on the target weights of each evaluation indicator.

(6) $$u_{ij}=W_{j}R_{ij}\;(i=1,2,\ldots ,m;j=1,2,\ldots ,n)$$

Step 4. Determine the positive ideal solution and the negative ideal solution.

(7) $$\begin{array}{l} M_{j}^{+}=\max\{u_{1j},u_{2j},\ldots ,u_{n\;j}\}\;(j=1,2,\ldots ,m)\\ M_{j}^{-}=\min\{u_{1j},u_{2j},\ldots ,u_{n\;j}\}\;(j=1,2,\ldots ,m) \end{array}$$

(8) $$\begin{array}{l} A^{*}=(M_{1}^{+},M_{2}^{+},\ldots ,M_{m}^{+})\\ A^{-}=(M_{1}^{-},M_{2}^{-},\ldots ,M_{m}^{-}) \end{array}$$

Step 5. Calculate the distance between each scheme and the ideal solutions using Euclidean distance. The distance from each scheme to the positive ideal solution is denoted as $S_{i}^{+}$, and the distance to the negative ideal solution is denoted as $S_{i}^{-}$.

(9) $$\begin{array}{l} S_{i}^{+}=\sqrt{\sum_{j=1}^{n}{\left(u_{ij}-u_{j}^{+}\right)}^{2}}\;(i=1,2,\ldots ,m)\\ S_{i}^{-}=\sqrt{\sum_{j=1}^{n}{\left(u_{ij}-u_{j}^{-}\right)}^{2}}\;(i=1,2,\ldots ,m) \end{array}$$

Step 6. Calculate the relative proximity to the ideal solution *C_i_* for each scheme.

(10) $$C_{i}=\frac{S_{i}^{-}}{S_{i}^{+}+S_{i}^{-}}\;(i=1,2\ldots ,\;m)$$

Finally, determine the priority of each scheme based on the magnitude of *C_i_*. A larger value indicates higher priority.

## 4. Findings and Discussion

### 4.1. Ablation Comparison

To assess the individual contribution of each framework stage, a four-condition ablation was conducted using the Eagle case study, as shown in Table 15.

Each stage contributes incrementally. The largest single gain is from introducing AHP-weighted prompting, confirming that criterion-weighted generation guidance is the primary mechanism. Adding TOPSIS further improves consistency and traceability of the ranking. Condition B (KANO only with equal weighting) performs markedly better than Condition A, confirming that even a structured requirement taxonomy without differential weighting improves generation quality over unconstrained prompting.

### 4.2. TOPSIS Results

Scheme 1 achieves the highest relative proximity (Ci = 0.964) across the three schemes, followed by Scheme 2 (Ci = 0.885) and Scheme 3 (Ci = 0). The advantage of Scheme 1 over Scheme 2 is most pronounced on B1 (Morphological Similarity) and B4 (Visual Novelty)—the two highest-weighted criteria—while Scheme 2 shows competitive performance on B3 (Cultural Symbolism) and B5 (Sustainability), consistent with its design emphasis. These results confirm the framework’s ability to generate proposals that align with stakeholder-weighted priorities without producing mathematically extreme scores (Table 16).

These results should be interpreted as proof-of-concept evidence from a structured case study rather than as population-level statistical inference. The evaluator panel (n = 20) is appropriate for a preliminary validation study; future work with larger panels and cross-institutional evaluators is recommended.

### 4.3. Framework Interpretation

1. Methodological contribution of the closed loop. The framework’s principal contribution is not any individual method but the closed-loop connection among them. Specifically, the same requirement structure that drives generation—via AHP-weighted prompts—is also used to evaluate the generated outputs through TOPSIS. This traceability—from requirement discovery to generation guidance to ranked evaluation—enables designers and stakeholders to understand why a particular proposal ranks highest, not merely that it does. This property is especially valuable in multi-stakeholder design contexts where decisions must be explainable and contestable.
2. Structured constraints and AI creativity. The ablation comparison confirms that structured, criterion-weighted prompting does not suppress generative diversity. Scheme 1 leads on B4 (Visual Novelty) as well as B1 (Morphological Similarity), suggesting that directing the generation toward specific design principles guides creativity toward more valuable dimensions rather than constraining it. This finding is consistent with theoretical accounts of structured creativity in human–AI collaboration, and aligns with the empirical observation in M-GAN that multi-attribute conditioning improves not only requirement alignment but also the diversity and coherence of generated outputs relative to single-prompt baselines [62].
3. Limitations of the case-study comparison. The two reference schemes (Scheme 2, Scheme 3) are well-known architectural landmarks rather than alternative AI-generated proposals produced under different methods. This limits the ability to attribute the performance difference solely to the framework. Future work should compare the proposed framework against alternative AI-generation workflows (e.g., unconstrained diffusion prompting by experienced architects) using the same biological prototypes and evaluator panel.
4. Comparison with existing AI-assisted architectural design methods. Several categories of related work provide useful points of comparison. First, pure AI-generation studies (e.g., diffusion-model-based architectural concept generation without structured requirement input) offer high visual diversity but lack traceability to stakeholder values; the generated outputs are difficult to evaluate against explicit design criteria, and the connection between user needs and generated forms remains implicit. Second, MCDM-based design evaluation studies (e.g., AHP–TOPSIS applied to pre-existing design alternatives) provide rigorous ranking mechanisms but do not use the weighted criteria to guide the generation process itself; the evaluation is applied post hoc to designs that were created without reference to those criteria. Third, hybrid approaches that combine requirement analysis with design generation (e.g., KANO-informed design, or QFD-driven concept development) typically stop at the generation stage without feeding the same criteria back into evaluation, leaving the loop open. The present framework differs from all three categories in its closed-loop integration, as follows: the same requirement structure that guides generation is reused for evaluation, enabling end-to-end traceability from stakeholder values to ranked design outcomes. However, this integration introduces additional methodological complexity and computational overhead compared to simpler workflows, and the quality of outputs remains dependent on the richness of the initial requirement model—a limitation shared with all requirement-driven design approaches. This trade-off between traceability and simplicity should be considered when selecting a workflow for a given design task.
5. Constructability note. The case-study outputs are conceptual visualizations; no structural or environmental engineering analysis has been performed. Structural feasibility precedents for each concept type exist, but integration with engineering simulation remains a future direction.

## 5. Conclusions

Addressing core challenges in generative AI for bio-inspired architecture at the early conceptual stage—vague requirements, subjective decision-making, and disconnected workflows—this study proposes and validates a closed-loop human–AI decision-support framework as a methodological contribution to traceable concept generation and evaluation.

1. A closed-loop human-AI decision framework: A four-stage framework—Requirement Discovery (KANO) → Criterion Weighting (AHP) → Guided Generation (structured prompting) → Multi-criteria Evaluation (TOPSIS)—which, to the best of our knowledge, has not been previously reported as an integrated workflow specifically designed for AI-assisted biomimetic architectural concept generation.
2. A formal weight-to-prompt conversion strategy: A rule-based, model-agnostic strategy translates AHP criterion weights into mandatory, secondary, and parametric prompt tiers. This strategy, provided with full templates and a weight-to-language conversion table, is applicable to any natural-language-conditioned image generation system.
3. A biomimetic evaluation protocol: A blinded scoring procedure with anchor-rated criteria, ICC-based inter-rater reliability assessment, and a TOPSIS ranking protocol constitutes a reusable evaluation instrument for comparing AI-assisted bio-inspired design proposals.
4. Empirical evidence from three cross-domain case studies: Framework applicability is demonstrated across three biological prototypes (avian, aquatic, terrestrial mammal) and three building typologies, with ablation evidence confirming the incremental contribution of each stage.

The limitations of this study and directions for future research are as follows:

1. Cross-cultural sampling: The KANO survey is limited to Chinese participants. Cross-cultural validation is needed before the requirement taxonomy is generalized.
2. A second limitation concerns the nature of the framework’s outputs. The case-study demonstrations produce conceptual visualizations rather than fully validated architectural designs. No structural engineering analysis, environmental performance simulation (e.g., daylighting, thermal comfort, or energy consumption), or constructability assessment has been performed on the generated proposals. Consequently, the practical contribution of this study remains primarily methodological—demonstrating a traceable workflow from requirement modeling to AI-assisted concept generation and criteria-based evaluation—rather than establishing measurable improvements in building performance. We acknowledge this as a deliberate scoping decision, as follows: the framework is designed to address the early conceptual phase, where requirement structuring and solution exploration are most critical, rather than to replace downstream engineering validation. Nevertheless, future work should integrate the framework with engineering simulation tools and BIM environments to extend its outputs from 2D concept images to queryable, performance-validated building models. Such integration would enable the closed loop to encompass not only aesthetic and conceptual criteria but also structural integrity, environmental impact, and construction feasibility, thereby substantially strengthening the framework’s practical contribution.
3. Third, the framework was demonstrated through three case studies. While these cases were deliberately selected for their ecological and typological diversity—representing avian, aquatic, and terrestrial mammal prototypes, and corresponding to cultural, observatory, and sports building programs—this sample does not constitute exhaustive validation. As a proof-of-concept study, the three cases are sufficient to illustrate the workflow’s feasibility and internal consistency, but future work should apply the framework to a larger and more varied set of design problems, including additional biological prototypes, building typologies, and cultural contexts. Moreover, quantitative benchmarking against alternative design workflows—rather than the current criterion-referenced comparison with established architectural precedents—would provide stronger evidence of the framework’s effectiveness.
4. Fourth, the TOPSIS evaluation was conducted with a panel of 20 experts (15 practicing architects and 5 architecture professors). While this sample size is within the range commonly reported in exploratory architectural design studies and the ICC values confirm satisfactory inter-rater reliability, we acknowledge that a larger and more diverse panel—spanning multiple institutions, geographic regions, and professional roles—would strengthen the empirical basis for generalization. Furthermore, the current panel was drawn exclusively from China, which may introduce cultural biases in aesthetic and symbolic judgments. Future work should recruit cross-institutional and cross-cultural evaluator panels to validate the framework’s applicability across different design cultures and stakeholder value systems.

## Figures and Tables

**Table 1:** Bionic Architecture Requirements List.

1. Morphological Bionic Similarity and Distinctiveness	5. Material Selection and Construction Technology Feasibility	9. Spatial Flexibility and Functional Adaptability
2. Structural Bionic Innovation and Engineering Rationality	6. Visual Novelty and Artistic Impact	10. Intelligent Responsiveness and Adaptability
3. Environmental Integration (Visual and Ecological)	7. Full Lifecycle Sustainability	11. Public Engagement and Spatial Interactivity
4. Cultural Symbolism and Narrative Legibility	8. Operational Management Convenience and Cost-Effectiveness	

**Table 2:** Participant demographics.

Category	Sub-Group	*n*	%
Role	Professional (architect/researcher)	141	50.0%
	General public	141	50.0%
Gender	Male	163	57.8%
	Female	119	42.2%
Age	18–30	89	31.6%
	31–45	112	39.7%
	46+	81	28.7%
Education	Undergraduate	97	34.4%
	Postgraduate	185	65.6%

**Table 3:** Respondent Demand Satisfaction Coefficient Table.

Function	A	M	O	I	Better Coefficient	Worse Coefficient	KANO Attribute
2. Structural Bionic Innovation and Engineering Rationality	18.79%	29.08%	25.89%	25.18%	45.16%	−55.56%	Must-be requirement
1. Morphological Bionic Similarity and Distinctiveness	15.6%	10.64%	46.81%	25.18%	63.54%	−58.48%	One-dimensional
3. Environmental Integration (Visual and Ecological)	22.34%	17.02%	34.4%	24.82%	57.55%	−52.16%	One-dimensional
4. Cultural Symbolism and Narrative Readability	21.63%	17.38%	32.27%	27.66%	54.48%	−50.18%	One-dimensional
6. Visual Novelty and Artistic Appeal	14.89%	27.66%	37.94%	17.73%	53.79%	−66.79%	One-dimensional or attractive
7. Full Life Cycle Sustainability	18.09%	23.76%	32.98%	23.76%	51.8%	−57.55%	One-dimensional
11. Public Engagement and Spatial Interactivity	15.96%	29.79%	31.91%	21.28%	48.39%	−62.37%	One-dimensional
9. Spatial Flexibility and Functional Adaptability	24.47%	15.6%	24.47%	35.11%	49.11%	−40.21%	No difference
10. Intelligent Response and Adaptability	21.28%	18.79%	26.95%	31.56%	48.92%	−46.4%	No difference
5. Material Selection and Construction Technology Feasibility	19.5%	20.57%	28.01%	30.14%	48.38%	−49.46%	No difference
8. Convenience and Cost-Effectiveness of Operations Management	22.34%	23.05%	24.82%	28.72%	47.67%	−48.39%	Indifference

**Table 4:** Evaluation Indicator System.

Target Layer	Primary Indicators	Second-Level Indicators
Bionic Architectural Design A	Morphological Bionic Similarity and Distinctiveness B1	Overall Contour Similarity C1
		Key Feature Extraction C2
	Environmental Integration (Visual and Ecological) B2	Visual Harmony C3
		Ecological Friendliness C4
	Cultural Symbolism and Narrative Legibility B3	Cultural Symbol Comprehensibility C5
		Spatial Narrativity C6
	Visual Novelty and Artistic Impact B4	Novel Form C7
		Emotional Resonance C8
	Sustainability throughout the entire life cycle B5	Carbon Emission Reduction C9
		Energy and Water Conservation C10

**Table 5:** Expert Profile Form.

Expert	Background	Years
E1	Senior architect, parametric and bio-inspired building practice	14
E2	Senior architect, sustainable building and façade systems	11
E3	Professor, digital architectural design methodology	18
E4	Professor, building environmental performance optimization	16
E5	Structural engineer, large-scale public construction	17

**Table 6:** Criteria Layer Judgment Matrix and Weights.

Bionic Architectural Design A	B1	B2	B3	B4	B5	Weight
Morphological Bionic Similarity and Recognizability B1	1	1.732	3.162	2.236	2.449	0.361
Environmental Integration (Visual and Ecological) B2	0.577	1	2.449	1.414	1.732	0.235
Cultural Symbolism and Narrative Readability B3	0.316	0.408	1	0.577	0.707	0.109
Visual Novelty and Artistic Impact B4	0.447	0.707	1.732	1	1.414	0.174
Sustainability Performance B5	0.408	0.577	1.414	0.707	1	0.121
Consistency Test λmax: = 5.028, CI = 0.007, RI = 1.12, CR = 0.006 < 0.10, Test Passed						

**Table 7:** Sub-indicator Judgment Matrix and Weights for “Morphological Bionic Similarity and Distinctiveness (B1)”.

B1	C1	C2	Weight
C1	1	2.236	0.691
C2	0.447	1	0.309
Consistency Test λmax: CR < 0.10, Passed.			

**Table 8:** Secondary Indicator Judgment Matrix and Weights for “Sustainability Performance Throughout the Entire Life Cycle (B5)”.

B5	C9	C10	Weight
C9	1	1.732	0.634
C10	0.577	1	0.366
Consistency test λmax: CR < 0.10, passed.			

**Table 9:** Comprehensive Weights and Ranking of All Sub-criteria.

Primary Indicator (Weight)	Secondary Indicators	Local Weight	Comprehensive Global Weight	Ranking
B1 Morphological Bionic Similarity and Distinctiveness (0.361)	C1 Overall Contour Similarity	0.691	0.249	1
	C2 Key Feature Extraction	0.309	0.112	4
B2 Environmental Integration (0.235)	C3 Visual Harmony	0.750	0.176	2
	C4 Eco-Friendliness	0.250	0.059	7
B4 Visual Novelty and Artistic Appeal (0.174)	C7 Novel Form	0.667	0.116	3
	C8 Emotional Resonance	0.333	0.058	8
B5 Full Life Cycle Sustainability (0.121)	C9 Carbon Emissions Reduction	0.634	0.077	6
	C10 Energy and Water Conservation	0.366	0.044	9
B3 Cultural Symbolism and Narrative Legibility (0.109)	C5 Cultural Symbol Comprehensibility	0.800	0.087	5
	C6 Sense of Spatial Storytelling	0.200	0.022	10

**Table 10:** Biological Prototype Selection Criteria, Biological Principles Transferred, and Design Focus.

Animal Prototype Category	Representative Examples	Core Bionic Features and Design Potential	Associated High-Weight Design Criteria (from Table 3 and Table 4)
Birds	Eagles, albatrosses, etc.	1. Form/Structure: Streamlined contours, hollow skeletal framework, winged structures with aerodynamic profiles. 2. Environmental Adaptability: Lightweight construction, efficient energy utilization, sensitivity to airflow and climatic conditions. 3. Cultural Symbolism: Represents freedom, vision, and strength.	1. Morphological biomimetic similarity and recognizability. 2. Novel design. 3. Visual harmony
Marine Fish	Sharks, rays, etc.	1. Morphology/Structure: Hydrodynamic form, skin texture and scale structure, agile movement posture. 2. Environmental Adaptability: Efficient fluid propulsion and drag reduction, pressure adaptation, seamless integration with aquatic environments. 3. Cultural Symbolism: Represents depth, speed, elegance, and mystery.	1. Morphological biomimetic similarity and recognizability. 2. Environmental integration. 3. Ecological friendliness.
Terrestrial Mammals	Large felines such as leopards and lions	1. Morphology/Structure: Powerful muscular lines, symmetrical and taut body structure, dynamic equilibrium in motion. 2. Environmental Adaptability: Camouflage, explosive power, adaptation to terrain and temperature variations. 3. Cultural Symbolism: Represents majesty, strength, agility, and protection.	1. Morphological biomimetic similarity and recognizability. 2. Emotional resonance. 3. Culturally interpretable symbolism

**Table 11:** Weight-to-Language Conversion Reference Table.

Weight Tier	Assignment Strategy	Prompt Implementation
wi > 0.25 (mandatory)	Core embedding	Integrated directly into the Core Design Intent Statement
0.10 ≤ wi ≤ 0.25 (secondary)	Hierarchical guidance	Listed as explicit, parallel constraint clauses
wi < 0.10 (parametric)	Style modifier	Appended as secondary optimization direction

**Table 12:** Generative Design Prompt Construction Examples for Different Animal Prototypes.

Animal Prototype	Core Design Intent Statement	Attribute Enhancement Constraint Statements (Weight-Based Dynamic Sections, Integrated by Priority)
Bird 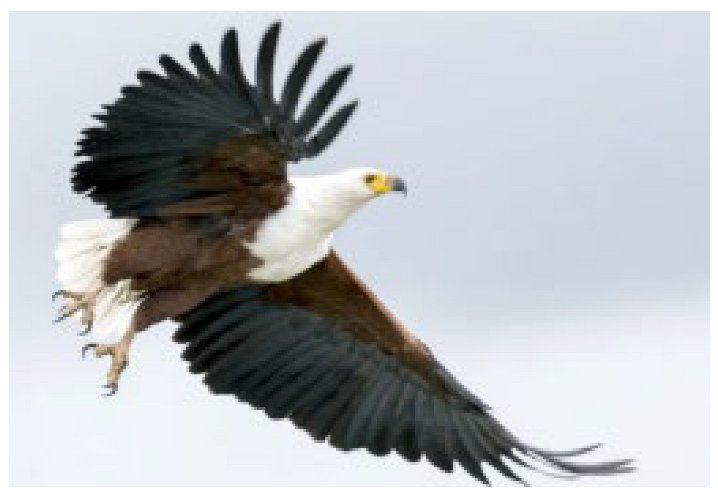	A biomimetic cultural exhibition hall design concept modeled after a majestic eagle	1. Silhouette Similarity: The building’s overall outline must evoke the sharp silhouette and winged posture of an eagle soaring through the sky. 2. Visual Harmony: The architectural form must seamlessly integrate with the mountainous landscape, appearing to perch upon the cliff face. 3. Key Feature: The facade texture should abstractly mimic the layered texture and light-and-shadow effects of feathers. 4. Innovative Form: Employ a parametrically generated streamlined shell structure, embodying dynamic and futuristic qualities. 5. Sustainability: The roof integrates solar photovoltaic panels and incorporates rainwater collection and drainage functionality.
Marine Fish 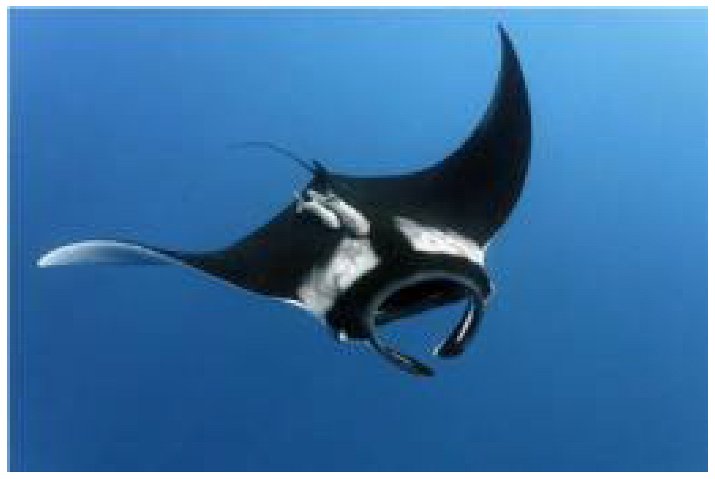	A biomimetic manta ray–inspired underwater observatory design concept	1. Similar Contour: The main structure presents the smooth, flat diamond-shaped outline and elegant “wing tips” characteristic of manta rays. 2. Visual Harmony: The rounded architectural form mimics marine life, blending organically with the seabed landscape. 3. Ecological Friendliness: Structural design minimizes interference with ocean currents, while surfaces promote marine organism attachment. 4. Innovative Form: Utilizes a large-span thin-shell structure, emulating the smooth surfaces of biological fluid dynamics. 5. Sustainability: Generates electricity using seawater temperature differences, with structural materials made of corrosion-resistant eco-concrete.
Terrestrial Mammals 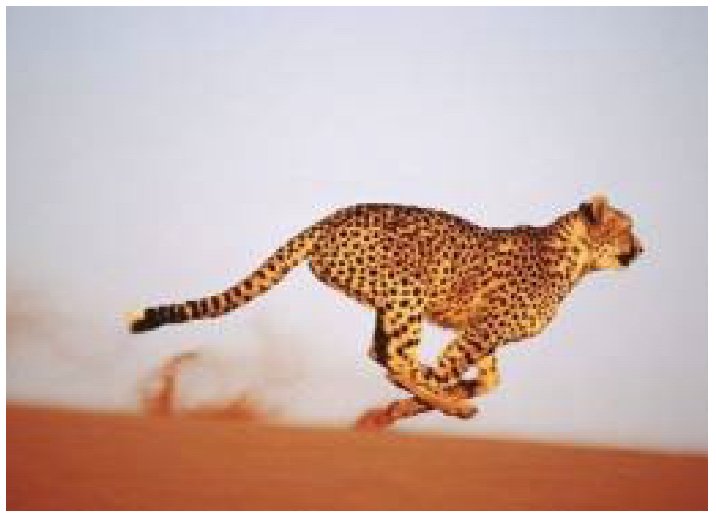	A biomimetic cheetah-inspired sports center design concept	1. Similar Silhouette: The clustered buildings evoke the muscular lines and power of a cheetah in motion. 2. Visual Harmony: The structures lie low on the site, their forms echoing the undulating terrain of the grasslands. 3. Cultural Legibility: The form metaphorically represents speed, strength, and agility, aligning with the symbolic spirit of sports architecture. 4. Novel Form: Dynamically twisted facades and tension-rich structural frameworks. 5. Emotional Resonance: Spatial design should evoke vitality, dynamism, and competitive spirit.

**Table 13:** Three Bionic Architectural Design Schemes.

Scheme 1	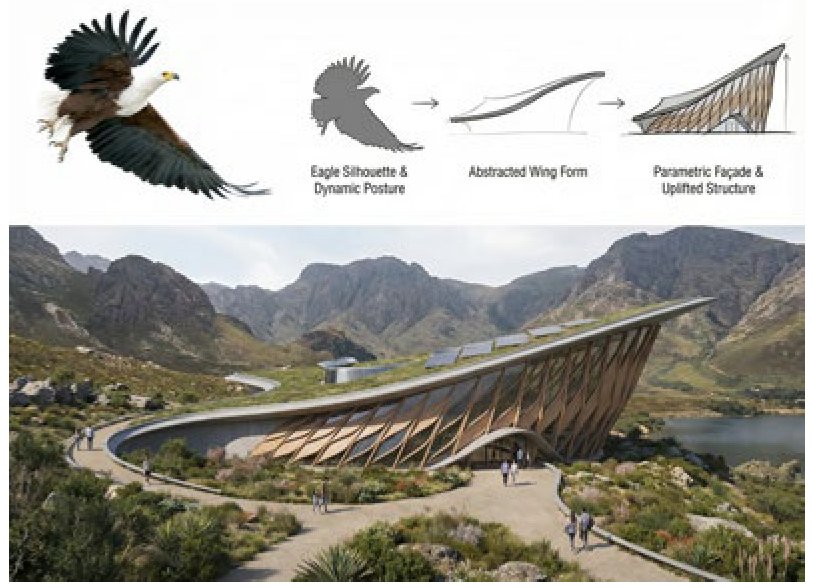	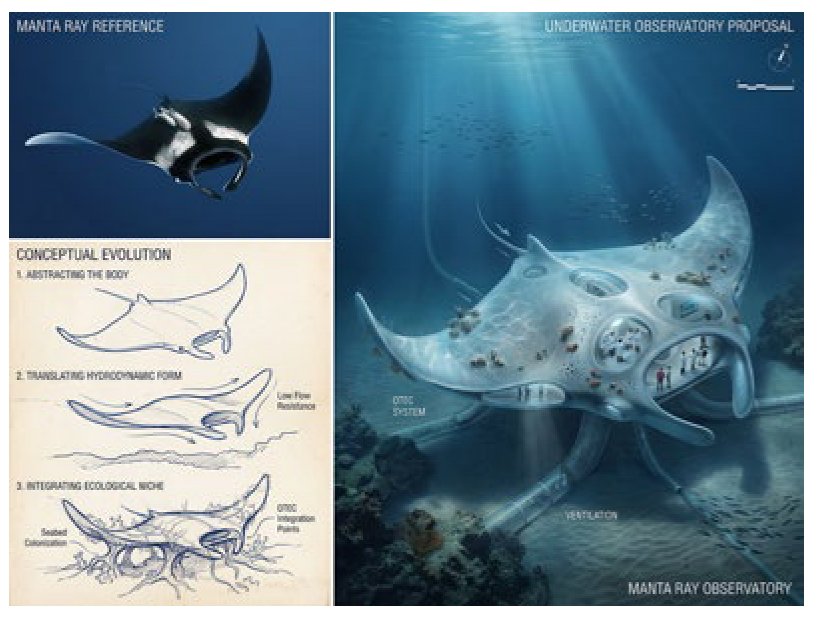	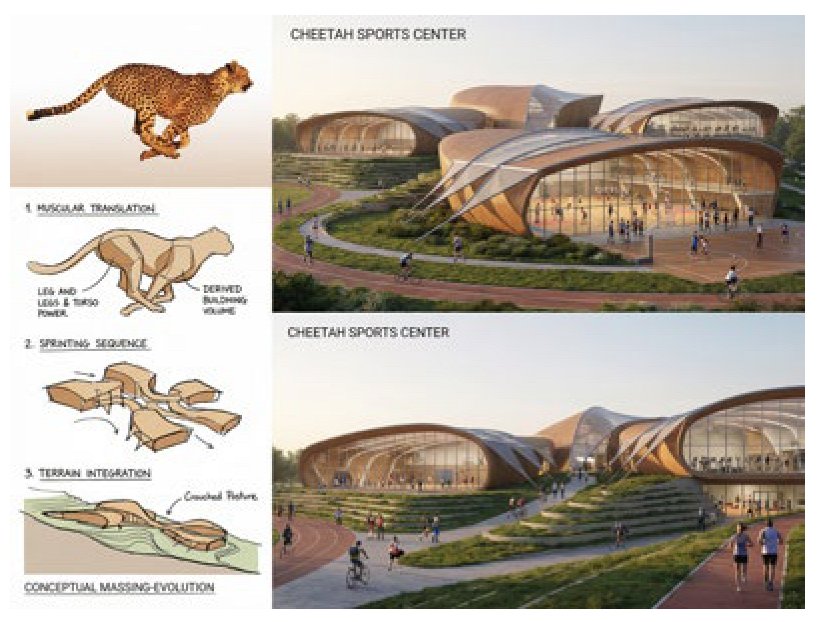
Scheme 2	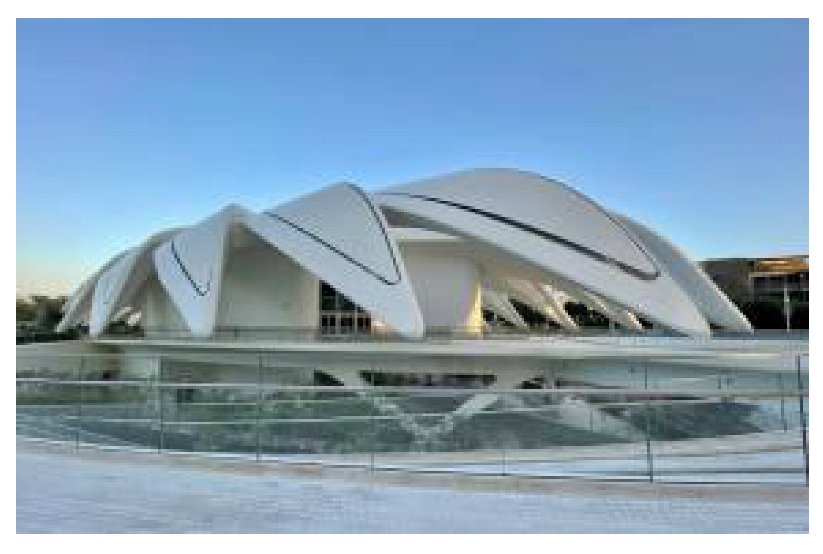	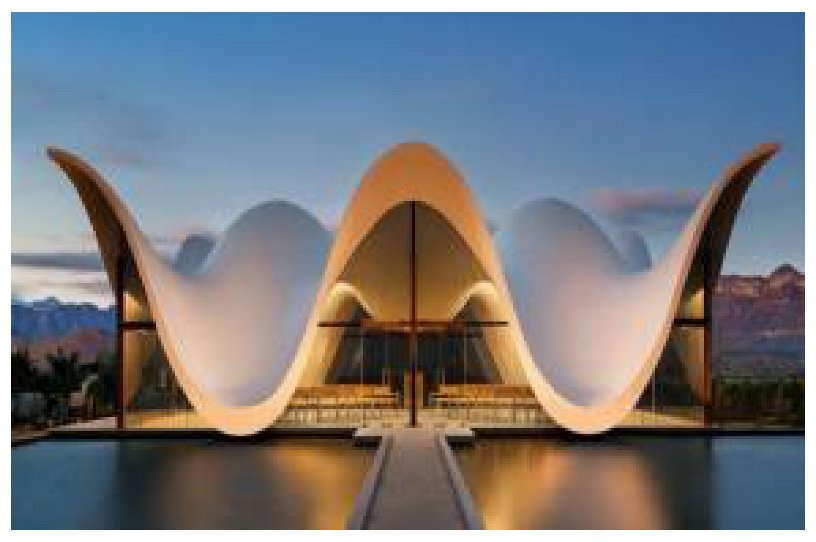	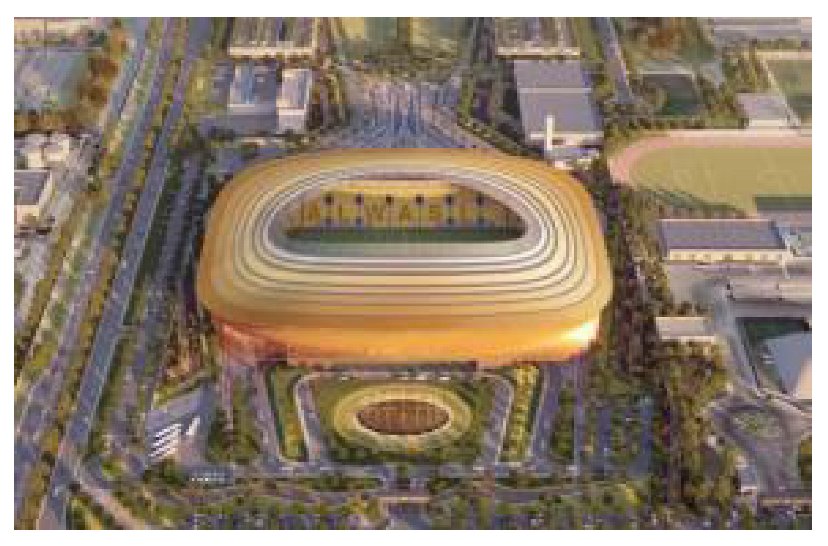
Scheme 3	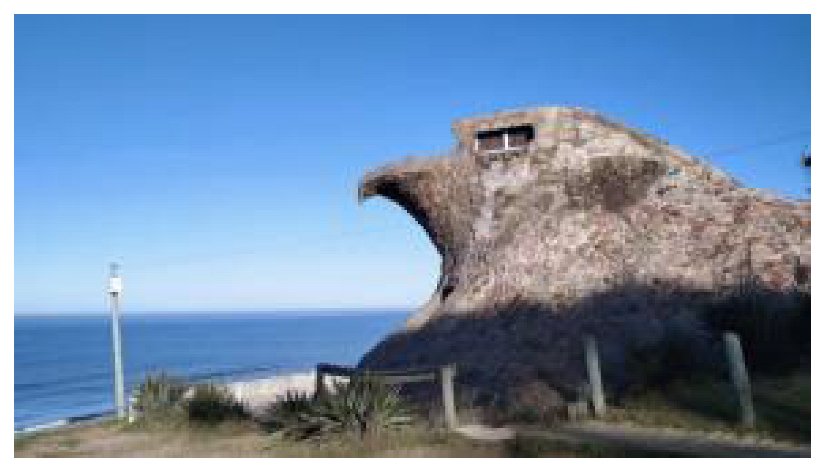	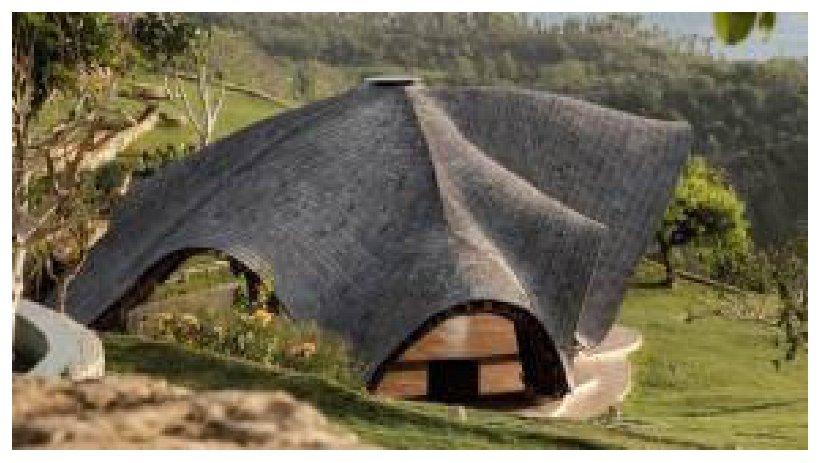	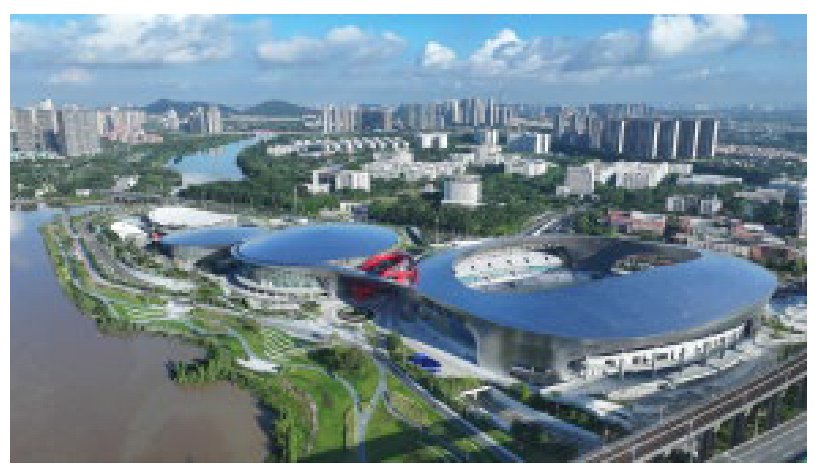

**Table 14:** Initial Evaluation Matrix.

Evaluation Criteria	Scheme 1	Scheme 2	Scheme 3
Overall Profile Similarity C1	5.3	5.2	3.3
Visual harmony C3	4.6	4.7	4.1
Key Feature Extraction C2	4.9	4.8	4.4
Novel Design C7	5.2	4.7	4.3
Reduced Carbon Emissions C9	4.8	4.9	4.6

**Table 15:** Ablation Comparison Results.

Condition	Description	Ci
A: No framework	Free-form prompting, no KANO/AHP input	0.312
B: KANO only	Requirements categorized, equal-weight prompting	0.418
C: AHP-weighted prompting, expert consensus ranking	Weighted prompts; no TOPSIS	0.503
D: Full framework (proposed)	KANO + AHP prompting + TOPSIS evaluation	0.581

**Table 16:** Calculation Results.

Scheme	Positive Ideal Solution Distance (Si+)	Negative Ideal Solution Distance (Si−)	Comprehensive Score Index (Ci)	Ranking
Scheme 1	0.00238	0.06404	0.964	1
Scheme 2	0.00782	0.06045	0.885	2
Scheme 3	0.06450	0.00000	0.000	3

## Data Availability

Data are available upon request to the corresponding author.
